# Apert's Syndrome

**DOI:** 10.5005/jp-journals-10005-1239

**Published:** 2014-04-26

**Authors:** Gudipaneni Ravi Kumar, Mandapati Jyothsna, Syed Basheer Ahmed, Ketham Reddy Sree Lakshmi

**Affiliations:** Assistant Professor, Department of Pedodontics and Preventive Dentistry Government Dental College and Hospital, Rims, Kadapa Andhra Pradesh, India; Assistant Professor, Department of Oral Pathology, Government Dental College and Hospital, Rims, Kadapa, Andhra Pradesh, India; Associate Professor, Department of Prosthodontics, Crown and Bridge, Government Dental College and Hopital, Rims, Kadapa, Andhra Pradesh India; Tutor, Department of Pedodontics and Preventive Dentistry Government Dental College and Hospital, Rims, Kadapa Andhra Pradesh, India

**Keywords:** Acrocephalosyndactyly, Craniosynostosis, Midface hypoplasia, Pseudocleft palate

## Abstract

Apert's syndrome (acrocephalosyndactyly) is a rare congenital disorder characterized by craniosynostosis, midfacial malforma­tion and symmetrical syndactyly of hands and feet. Craniofacial deformities include cone-shaped calvarium, fat forehead, prop-tosis, hypertelorism and short nose with a bulbous tip. Intraoral findings include high arched palate with pseudocleft, maxillary transverse and sagittal hypoplasia with concomitant dental crowding, skeletal and dental anterior open bite and several retained primary teeth. We report one such case of 14-year-old boy having all the classical features of Apert's syndrome with particular emphasis on brief review of genetic features.

**How to cite this article: **Kumar GR, Jyothsna M, Ahmed SB, Lakshmi KRS. Apert's Syndrome. Int J Clin Pediatr Dent 2014;7(1):69-72.

## INTRODUCTION

Apert^1^ in 1906 reported nine such cases and since then his name has been associated with acrocephalosyndactyly. It is characterized by premature fusion of cranial sutures (cranio-synostosis) which restricts the growth, leading to craniofacial abnormalities. These patients have frontal bossing, narrow high arched palate, midfacial malformations and symmetri­cal syndactyly of both hands and feet with broad and short fused nails. Mental retardation is usually present but its true incidence is not known.^2^ According to Cohen,^3^ the incidence of Apert's syndrome is about 15 per 1,000,000 live births. Apert's syndrome has been rarely reported from India.^4^ We present one such case of Apert's syndrome.

## CASE REPORT

A 14-year-old boy reported to the Department of Pediatric Dentistry, Govt Dental College and Hospital, Rajiv Gandhi Institute of Medical Sciences, Kadapa, with the chief com­plaints of tooth mobility and pain in lower right front tooth. The patient presented with unusual craniofacial and dental features which prompted a further detailed examination of the case. A provisional diagnosis of Apert's syndrome was established, and detailed examination has been carried out.

The boy was fourth in the family born to non-consan­guineous parents after the normal labor. The birth history of the patient was uneventful with no known exposure to infection, drugs or irradiation during his mother's pregnancy. No similar malformations were known in both parent's family. Both parents and patient's siblings were found to be normal on clinical and radiological examination.

**Fig. 1:**
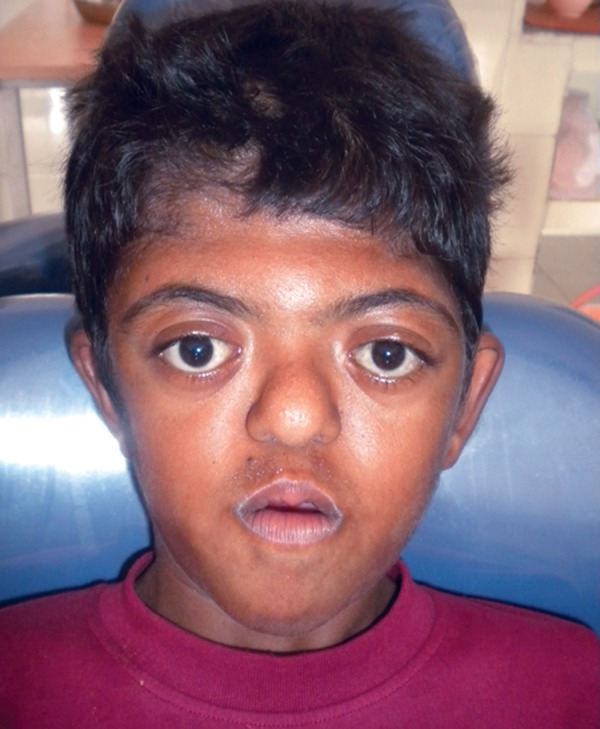
Frontal view of the face; midface hypoplasia, depression of nasal bridge, proptosis, hypertelorism

**Fig. 2:**
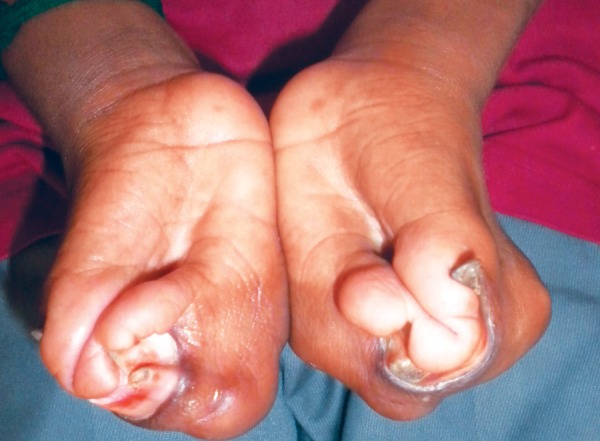
Syndactyly of hands

**Figs 3A and B:**
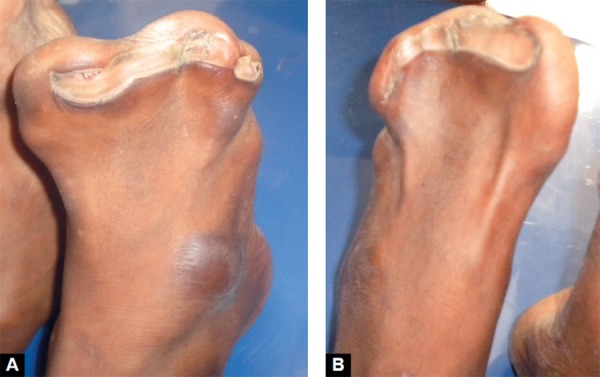
(A) Syndactyly of right foot and (B) syndactyly of left foot

Extraoral examination revealed abnormal shape and contour of the head (turribrachycephaly), depression of the nasal bridge, frontal bossing, midface hypoplasia, charac­teristic ‘crossbow shape’ of upper lip, trapezoidal mouth-shape and cephalometric dolichofacial pattern, proptosis and exorbitism (Fig. 1). Examination of the upper limbs showed symmetric soft tissue syndactyly of all digits almost forming a single unit with a single broad fused nail causing a spoon-like deformity (Fig. 2). The lower limbs also showed symmetrical syndactyly of all toes with broad fused single nail (Figs 3A and B), delayed milestones, and mild mental deficiency was also recorded. Other systemic examination revealed no abnormality.

**Fig. 4:**
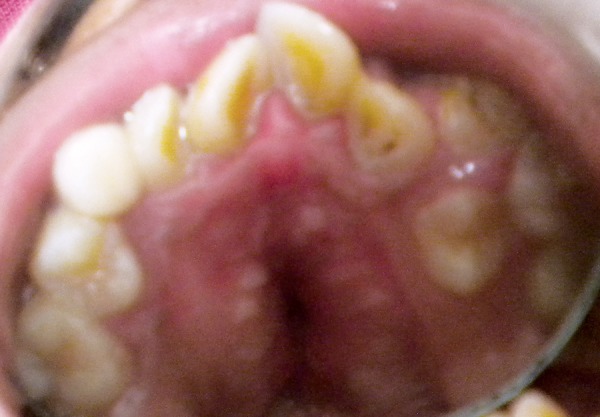
Pseudocleft

**Fig. 5:**
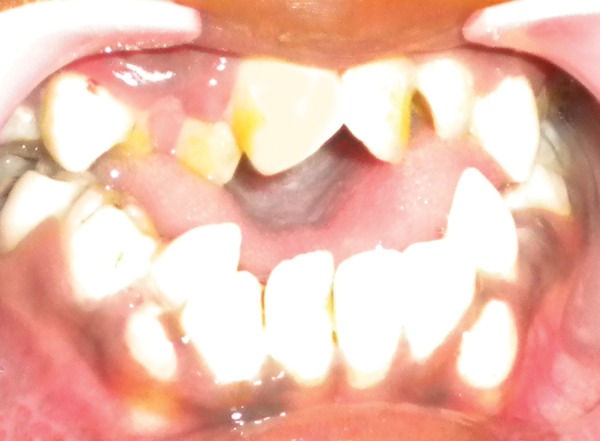
Intraoral view

The intraoral features include high-arched palate asso­ciated with lateral swellings of the palatine processes on either side of the midline mimicking a ‘pseudocleft’ (Fig. 4). The oral hygiene was poor with abundant dental plaque and calculus. The gingiva showed congestion and infammatory enlargement in relation to anterior teeth with several decayed teeth. Angle's pseudo class III malocclusion with severe crowding of the maxillary and mandibular anterior teeth with skeletal anterior open bite and posterior cross bite was observed. The primary teeth were retained with delayed and ectopic eruption of permanent teeth (Fig. 5).

**Figs 6A and B:**
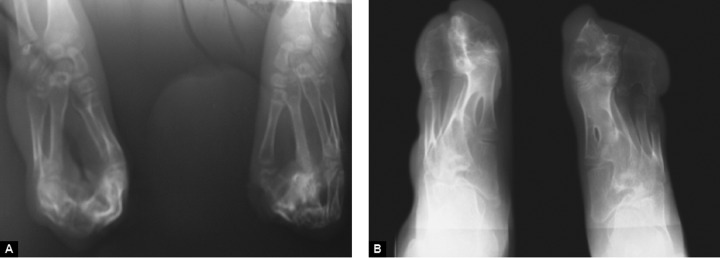
(A) Radiographs of hand and (B) radiographs of feet

**Figs 7A and B:**
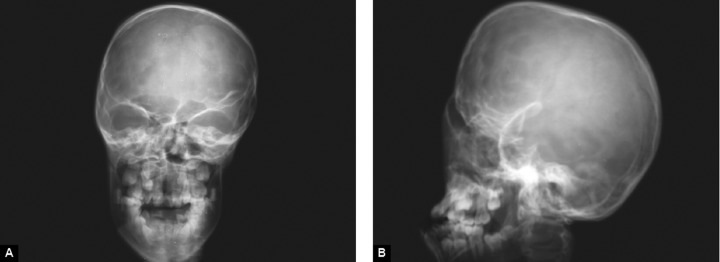
(A) Anteroposterior view of skull and (B) lateral view of skull

Radiographs of both hands showed complete bony syn-dactyly of all the digits involving the phalanges (Fig. 6A). Radiographs of right foot showed syndactyly of all phalanges with fusion of first and second metatarsals and left foot showed fusion of first and second phalanges at their distal ends along with the fusion of first and second metatarsals, the third, fourth and ffth phalanges were fused at their proximal ends (Fig. 6B). Skull radiographs revealed fused coronal sutures, turribrachycephalic skull contour and elongated fat forehead (Figs 7A and B).

## DISCUSSION

Apert's syndrome is an autosomal dominant disorder but, in many cases, the inheritance is sporadic.^5^ An association between this condition and a high parental age has been suggested. The risk of a second child being affected is 1%.^6^Most of the molecularly characterized cases of Apert's syndrome result from two specific mutations of a gene 10q26 located on chromosome, encoding fibroblast growth factor receptor 2 (FGFR-2). The two mutations involve C-to-G transversions at adjacent codons in exon IIIa of the gene. The first mutation is C934G transversion, leading to a change of codon TCG to TGG, producing a serine-to-tryptophan substitution at amino mutation acid 252 (S252W or Ser252Trp). The second is a C937G transversion, changing codon CCT to CGT, resulting in proline to arginine substitution at amino acid 253 (P253R or Pro253Arg).^7^ The former (S252W) is the most common mutation, occurring in 67% of patients and has been proposed to be asso­ciated with more severe craniofacial anomalies, whereas the later (P253R) may be associated with more severe syndactyly.^8^ These mutations affect the region linking the immunoglobulin-like domains II and III of FGFR-2 and resulting in increased affinity and altered specificity of ligand binding.^9^ This in turn leads to deregulation of cell migration, proliferation and differentiation and ultimately to premature osteogenesis and skeletal abnormalities that characterize the syndrome. The syndactyly is marked and resem bles a ‘mitten hand’ or ‘sock foot’ there is usually a complete fusion of the distal soft tissues and occasionally of the bones. The thumbs and big toes may or may not be involved in the fusion. Other less fre quent skeletal anomalies may be present. In the same category of major craniosynostosis syndromes associated with a mutation of the FGFR family apart from Apert's syndrome, other well-defined clinical entities are: Crouzon syndrome, Jackson-Weiss syndrome and Pfeiffer syndrome.^10^

Apert's syndrome cases were usually present with abnormalities of the lower respiratory tract includes choanal stenosis, anomaly of the tracheal cartilage and some degree of air obstruction (40% of cases ).^11^ Cardiovascular and genitourinary defects occur in 10 and 9.6% of patients with Apert's syndrome respectively.^12^ The literature also reports of skin manifestations in Apert's syndrome, such as acne, hyperhydrosis, hypopigmentation and hyperkeratosis of plantar surfaces.^13^ Fortunately, no other systemic abnormalities were found in this patient apart from mild mental deficiency.

A complex treatment plan involving prophylactic and therapeutic approach was formulated for the patient which includes regular mechanical and chemical professional plaque control, with fluoride and chlorhexidine applications to control the intense carioactivity and periodontal infa-mmation. A thorough periodontal examination involving pseudopocket depths and attachment loss were recorded. The professional plaque control including supragingival debridement was performed. The existing caries lesions were restored and orthodontic treatment was planned to treat the malocclusion and lip position. The retained primary teeth were extracted after obtaining the physician consent.

The treatment of Apert's syndrome should ideally begins at birth with proper diagnosis and these patients generally require lifelong management by a team of healthcare specialists. The therapeutic management of children with Apert's syndrome should be multidisciplinary in approach which includes surgical correction of the craniosynostosis, midfacial hypoplasia and syndactyly. Prenatal sonographic detection of structural abnormalities associated with Apert's syndrome is usually possible. The specialist should inform the parents that prognosis is not optimal (increased risk of mental retardations and multiple postnatal operations) so that the parents could opt for termination of pregnancy before the stage of fetal viability.^14^ Nonsurgical manipulation of Apert's syndrome may be a possibility in the future, by using selective inhibitors of the FGFR-kinase domain.^15^

## CONCLUSION

Craniosynostosis which occurs in sporadic and hereditary forms remains as a major medical condition with considerable morbidity. Dental professionals should be well informed on the oral aspects of patients with Apert's syndrome to render the effective dental needs and reassure the psychological status of both the child and their parents.
